# A systematic review of phenotypic and epigenetic clocks used for aging and mortality quantification in humans

**DOI:** 10.18632/aging.206098

**Published:** 2024-08-30

**Authors:** Brandon Warner, Edward Ratner, Anirban Datta, Amaury Lendasse

**Affiliations:** 1 Verseon International Corp., Fremont, CA 94538, USA; 2 Department of IST, University of Houston, Houston, TX 77004, USA; 3 Department of Engineering Management and Systems Engineering, Missouri University of Science and Technology, Rolla, MO 65409, USA

**Keywords:** aging, DNA methylation, epigenetics, machine learning, biomarkers

## Abstract

Aging is the leading driver of disease in humans and has profound impacts on mortality. Biological clocks are used to measure the aging process in the hopes of identifying possible interventions. Biological clocks may be categorized as phenotypic or epigenetic, where phenotypic clocks use easily measurable clinical biomarkers and epigenetic clocks use cellular methylation data. In recent years, methylation clocks have attained phenomenal performance when predicting chronological age and have been linked to various age-related diseases. Additionally, phenotypic clocks have been proven to be able to predict mortality better than chronological age, providing intracellular insights into the aging process. This review aimed to systematically survey all proposed epigenetic and phenotypic clocks to date, excluding mitotic clocks (i.e., cancer risk clocks) and those that were modeled using non-human samples. We reported the predictive performance of 33 clocks and outlined the statistical or machine learning techniques used. We also reported the most influential clinical measurements used in the included phenotypic clocks. Our findings provide a systematic reporting of the last decade of biological clock research and indicate possible avenues for future research.

## INTRODUCTION

Aging is the most influential risk factor for many disease states [1]. Developing interventions in the aging process will require building a systematic understanding of the underlying causal factors and associated biomarkers and epigenetic markers that lead to biological and cellular deterioration. The nine hallmarks of aging are key frameworks for describing such phenomena [2]. Epigenetic alterations, one of the nine hallmarks, can be accurately measured using DNA methylation (DNAm) levels [3, 39]. DNAm is the process in which a methyl group is added to the 5’ position on cytosines in cystine guanine dinucleotides, or CpGs [4]. Epigenetic clocks predict one’s cellular age by measuring this process of epigenetic deterioration using methylation data [5] and have been shown to predict chronological age with a correlation of 0.96 or higher [6]. Since then, many other comparable epigenetic clocks have been proposed using varying CpG sites, cohorts, and algorithmic approaches.

Phenotypic clocks are an alternative approach to measuring age-related deterioration and mortality. Phenotypic clocks use easily measurable biological and physiological clinical biomarkers to quantify aging and disease-related mortality (i.e., “aging scores”) and have been shown to predict mortality more accurately than chronological age [7–14]. Phenotypic clocks are easier to model when compared to epigenetic clocks because they use readily available measurements collected in a standard clinical setting. Additionally, they may provide insights into intracellular phenomena, while epigenetic clocks only measure at the cellular level. Further, changes in lifestyle, such as diet or exercise, are more readily manifested in alterations in these clinical biomarkers, providing valuable feedback that may be actionable.

This study aims to comprehensively survey existing research on epigenetic and phenotypic clocks. This survey extends previous systematic reviews and meta-analyses on epigenetic clocks [15, 16] by including recent epigenetic clocks using artificial neural networks, as well as providing greater focus on phenotypic clocks. To achieve these goals, this study conducted an extensive systematic review of all epigenetic and phenotypic age measurement literature, the first study of its kind. This study fills a critical gap in the literature by synthesizing studies on epigenetic clocks and phenotypic clocks, with a focus on the clinical utility of each.

## METHODS

This systematic review was designed in accordance with the Preferred Reporting Items for Systematic Reviews and Meta-Analyses (PRISMA) guidelines for protocol, search strategy, and risk of bias assessment [17].

### Search strategy

**Figure 1:**
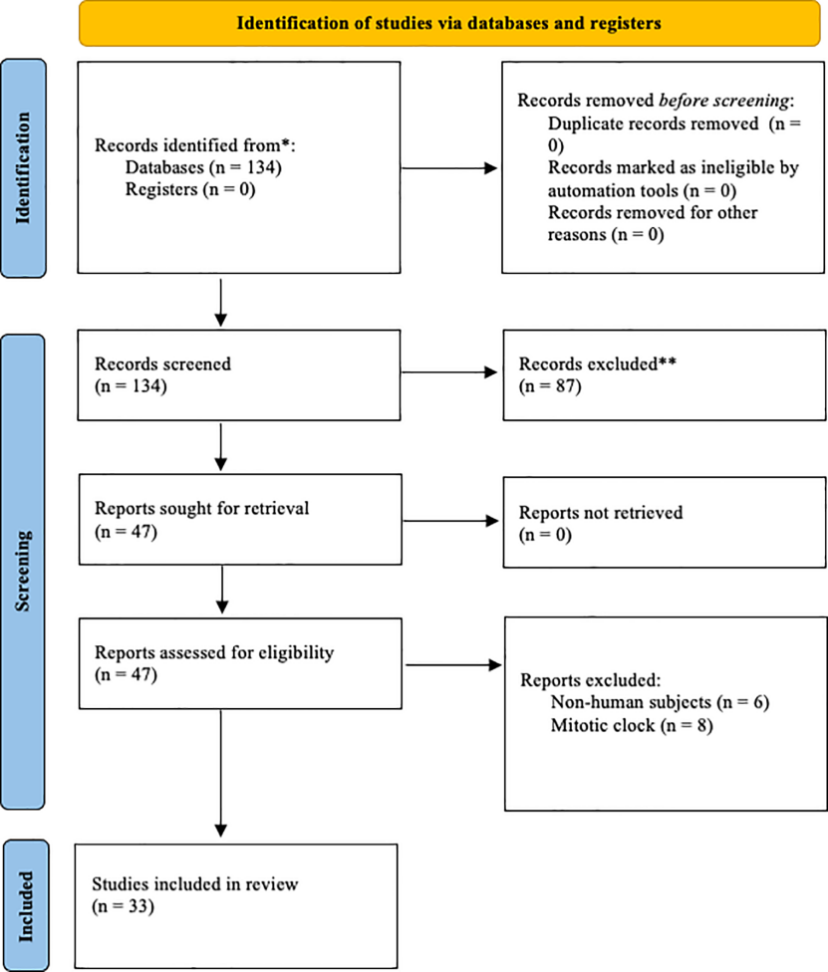
Prisma diagram illustrating the search process and exclusion criteria.

A comprehensive literature search was performed on June 8, 2023, and was conducted using the PubMed online database. Additionally, a grey literature (i.e., citation tracing) and Google Scholar search were conducted to ensure optimal coverage of other journals and preprint publications. PubMed search terms included ‘epigenetic clock’ OR ‘biomarker clock’ AND aging, cellular (MeSH Terms) OR dna methylation (MeSH Terms) OR methylation, dna (MeSH Terms) OR longevity (MeSH Terms) AND biomarkers (MeSH Terms) OR ‘phenotypic’. After the search was complete, resources were screened according to the inclusion criteria outlined in the following section. A visual representation of the search strategy is shown in Figure 1.

### Inclusion and exclusion criteria

The included articles were limited to primary studies (i.e., non-reviews) available in the English language that concerned human subjects. We included clocks that used human blood or saliva samples to predict chronological age, phenotypic age, or mortality risk. We did not include mitotic clocks used to predict cancer risk and progression since this is outside the scope of this review. Furthermore, we excluded papers that were not primary studies (i.e., papers that reproduced and compared already published clocks).

### Data extraction

Studies that fit the inclusion criteria were analyzed according to various descriptive statistics provided in the original literature. These included the publication year, type of clock, number of CpGs or blood biomarkers used, number of subjects, and accuracy of prediction (r). Additionally, we extracted model coefficients for the most influential plasma biomarkers used in phenotypic clocks.

## RESULTS

### Study selection

The initial search resulted in 134 articles. After abstract screening, 87 articles were excluded. After full-text screening, 14 articles were removed. This yielded 33 included studies. Included studies featured a variety of biological clocks, including methylation-based clocks, mortality clocks, and phenotypic clocks. Table 1 presents the characteristics and performance of all the included clocks in our study.

**Table 1:** Descriptive statistics of all included first-generation epigenetic clocks.

**References**	**# of CpGs**	***n* subjects**	**Performance (r)**
Horvath et al. (2020) [18]	36,000	850	0.990
Q. Zhang et al. (2019) [19]	514	13,566	0.990
de Lima Camillo et al. (2022) [20]	20,318	8,050	0.980
Galkin et al. (2021) [21]	24,538	1,293	0.980
Vidaki et al. (2017) [22]	16	1156	0.980
Correia Dias et al. (2020) [23]	4	53	0.977
Bekaert et al. (2015) [24]	4	206	0.973
Lee et al. 2020) [25]	1791	2,227	0.970
Thong et al. (2021) [26]	3	196	0.969
Levy et al. (2020) [27]	300,000	503	0.960
X. Li et al. (2018) [28]	6	539	0.960
Horvath et al. (2018) [29]	391	3931	0.960
Horvath, (2013) [30]	353	3,931	0.960
Han et al. (2020) [31]	9	973	0.943
Weidner et al. (2014) [32]	99	656	0.933
Garagnani et al. (2012) [33]	1	64	0.920
Hannum et al. (2013) [34]	71	482	0.905
Freire-Aradas et al. (2018) [35]	6	180	0.893
Florath et al. (2014) [36]	17	249	0.880
Koch and Wagner (2011) [37]	5	130	0.825
Vidal-Bralo et al. (2016) [38]	8	390	0.775
Naue et al. (2017) [43]	13	208	NR

Accuracy (r) represents the Pearson’s correlation coefficient of the predicted ages with the true ages in the validation set. NR stands for ‘not reported.’ Clocks are sorted by performance in descending order.

### Epigenetic clocks

Epigenetic clocks generally follow the standard protocol for regression problems. After data acquisition, one may conduct feature (i.e., CpG) selection and/or dimensionality reduction approaches in hopes of optimizing the prediction of the output (in this case, chronological age). The Pearson correlation coefficient of the predicted age and the “true” chronological age is the most common metric used in the literature to measure such performance. Below, we outline the performance of all included epigenetic clocks along with their respective modeling statistics, including the number of CpGs and the number of subjects. In some cases, the authors of the original literature chose a different validation metric. In these cases, we denoted their performance as “not reported” (NR). In the results below, we separate our epigenetic clock findings into three categories: first-generation, second-generation, and third-generation. We do so because each of these types of approaches are fundamentally different from the others and a direct comparison would not be appropriate.

### First-generation clocks

Epigenetic clocks may be divided into several distinct generations. First-generation clocks rely on cross-sectional data alone to investigate the association of biomarkers with chronological age. In these approaches, deviations between the true chronological age and the predicted age are treated as indications of accelerated or decelerated aging. Horvath’s first epigenetic clock (Horvath, 2013) inspired a wealth of research into this type of approach and many studies have substantiated this hypothesis by confirming that accelerated epigenetic aging is associated with various deleterious phenotypes, including post-traumatic stress (Boks et al., 2015), obesity (Horvath et al., 2014), and more. Additionally, increased epigenetic age has been shown to predict mortality later in life (Marioni et al., 2015), albeit moderately. We present a collection of characteristics and prediction performances of first-generation clocks below.

### Second-generation clocks

While first-generation clocks made great progress in understanding the mechanistic properties of cellular aging, various drawbacks are inherent to the chronological age approach. First, Horvath, and Raj (2018) concluded that first-generation clocks are only able to provide weak associations with physiological measures of dysregulation. Secondly, and perhaps most critical, is the paradox of chronological age. Zhang et al. (2018) found that DNAm levels can, theoretically, provide perfect chronological age predictions if enough data is available, but useful mortality and phenotypic associations attenuate as predictions near perfection. Consequently, second-generation clocks were proposed to address these concerns. Rather than using candidate biomarkers to predict chronological age, second-generation clocks investigate the association of biomarkers with time-to-event data, specifically time-to-mortality. The most influential second-generation clocks include PhenoAge (Levine, 2018), GrimAge (Lu et al., 2018), and MetaboHealth (Deelin et al., 2019). Each of these approaches used very different strategies to predict mortality risk. PhenoAge used calendar age and 9 clinical measurements to predict phenotypic age, which was then used to regress on DNAm levels to identify 513 CpG sites that influence disease and mortality among those of the same calendar age. GrimAge used 12 plasma proteins and smoking pack-years regressed on all-cause mortality, identifying 1030 influential CpG sites. Lastly, MetaboHealth used metabolic predictors to identify 14 biomarkers independently associated with all-cause mortality. Each of these approaches exhibited greater strengths of association with all-cause mortality than first-generation clocks.

### Third-generation clocks

Third-generation clocks are characterized by the use of longitudinal data to predict aging rates. The most noteworthy third-generation clock is DunedinPoAm [36], which measured longitudinal changes of 18 clinical biomarkers to predict rates of aging. Like second-generation clocks, DundeinPoAm exhibited superior mortality risk prediction than first-generation clocks. A few other longitudinal studies have been conducted using methylation data [93–95]. As longitudinal data becomes more readily available, third-generation clocks will become more prevalent due to their predictive power.

### First-generation clock modelling decisions and performance

Unsurprisingly, there is a wide range in predictive performance of the various clocks due to heterogeneous data sources. The clocks that featured the highest correlation with chronological age were [18, 19], with Pearson correlations of 0.990 with the output. Interestingly, Horvath’s clock used 36,000 CpG sites in the model, significantly more than the number of training samples. The authors did so by employing feature selection methods based on model coefficients extracted from linear models. The Horvath clock [18] used penalized regression models, while the Zhang clock [19] used elastic net regression. [20–22] attained the next best correlated predictions (R = 0.98) and were all based on artificial neural networks (ANNs). [22] built multiple ANNs, including multi-layer perceptrons (MLPs), radial bias functions (RBFs), probabilistic neural networks (PNNs), and generalized regression neural networks (GRNNs). Both [20] and [22] built deep learning models, but [20] paired their model with SHAP (Shapley Additive Explanations) [92] values to provide interpretability.

### Phenotypic clocks

While biological clocks have focused more on methylation data in recent years, phenotypic clocks also provide valuable longevity estimations using readily available clinical measurements. To that end, phenotypic clocks have been proven to predict mortality more accurately than chronological age in a variety of scenarios [8–14, 35].

Klemera and Doubal [40] were the first to prove that biological age estimates using purely clinical values provided more robust measurements of mortality than chronological age. Since then, phenotypic models have largely focused on using proportional hazard and survival models such as the Gompertz mortality model [10, 41]. These models often use chronological age along with other biomarkers of aging to predict mortality. [40] were the first to use chronological age as a biomarker and anticipated this would be viewed as controversial due to the heterogeneity of aging processes among different people.

Before Klemera and Doubal’s method, most phenotypic models fell under three categories: multiple linear regression (MLR), principal component analysis (PCA), or Hochschild’s method [42]. MLR models choose biomarkers according to their correlation with chronological age and were established by Hollingsworth [43] and others. MLR models are simple to implement but distort the biological age at the regression edge (i.e., at the youngest and oldest ages). PCA-based biological clocks avoid distortion at the regression edge but cannot avoid the paradox of chronological age [44]. Hochschild’s method solves the paradox of chronological age but is nonstandard and somewhat complex to implement. Klemera and Doubal’s method improves on each of these methods by solving the paradox of aging by minimizing the distance between regression lines for each biomarker point, providing a better estimation of mortality than chronological age. [50] evaluated multiple variations of both the Klemera and Doubal phenotypic clock and frailty indices [78–79] and evaluated their performance both with and without chronological age as an input variable. The authors found that the models without chronological age input captured the most variability of mortality indicators, though more research is needed on this subject.

Unlike epigenetic clocks, phenotypic clocks may be modeled using a variety of approaches. Klemera and Doubal’s popular approach uses chronological age as one of the input biomarkers, along with other standard biomarkers such as blood glucose. However, using chronological age to calculate biological age is a somewhat controversial modeling decision. This can, as expected, lead to a very high correlation, as shown in Table 2.

**Table 2:** Descriptive statistics of phenotypic clocks that use chronological age as input.

**References**	**Output variable**	**# biomarkers**	***n* subjects**	**Performance (r)**
Chen et al. (2023) [45]	Chronological	12	12,377	0.980
Liu et al. (2018) [41]	Chronological	13	11,432	0.960
Levine (2013) [9]	Chronological	13	9,389	NR

Accuracy (r) represents the Pearson’s correlation coefficient of the predicted ages with the true ages in the validation set. NR stands for ‘not reported.’ Clocks are sorted by performance in descending order.

There are, however, phenotypic clocks that do not use chronological age as input to the model. A variety of modeling structures have been employed to calculate phenotypic age in this way. Putin et al. (2016) used an ensemble of deep neural networks, while Husted et al. (2022) and Park et al. (2009) used principal component analysis (PCA) approaches. [14] used a very different approach, employing agglomerative clustering to determine influential biomarkers in aging and mortality processes. The performance and descriptive statistics of each of these models are shown in Table 3.

**Table 3:** Descriptive statistics of phenotypic clocks that do not use chronological age as input.

**Reference**	**Output variable**	**# biomarkers**	***n* subjects**	**Performance (r)**
Putin et al. (2016) [47]	Chronological	41	62,419	0.910
Husted et al. (2022) [48]	Chronological	9	100	0.86
Park et al. (2009) [46]	Chronological	11	1588	0.762
Nakamura and Miyao, (2007) [49]	Chronological	5	86	0.720
Sebastiani et al. (2017) [14]	N/A (unsupervised clustering)	19	4704	NR

Accuracy (r) represents the Pearson’s correlation coefficient of the predicted ages with the true ages in the validation set. NR stands for ‘not reported.’ Clocks are sorted by performance in descending order.

Despite widespread use in epigenetic clocks, artificial neural networks have, to our knowledge, only been employed in one phenotypic clock. [47] used an ensemble of 21 deep neural networks (DNNs) of varying structure and depth to predict chronological age using physiological biomarkers alone. Furthermore, the authors paired their model with a feature importance wrapper-based strategy called Permutation Feature Importance (PFI), which allowed the authors to ascertain which variables are most influential in the model. The authors attained impressive prediction performance (r = 0.91), but the dataset used in the experiments is not open-source and, thus, is not reproducible.

### Biomarker importance in phenotypic clocks

Many phenotypic clocks are modeled using linear models due to their ease of interpretability. Unlike artificial neural networks, information from linear models can be directly extracted from coefficients in the model. These coefficients measure the relative importance of each feature in the model and can be used to better understand the model’s predictions. Many phenotypic clocks identified the same plasma biomarkers as most influential in the aging process. A brief analysis of model coefficients (i.e., feature contribution) used in phenotypic clocks was conducted to identify which plasma biomarkers were consistently found to be influential in primary literature. The varying magnitude of the coefficients can be attributed to the other features included in each of the models. The results of this analysis are reported in Table 4.

**Table 4:** Clinical plasma biomarkers and their respective regression coefficients.

**Biomarker**	**Nakamura and Miyao, (2007) [49]**	**Levine, (2013) [10]**	**Mitnitski et al., (2017) [50]**	**Liu et al., (2018) [41]**
Systolic blood pressure	0.580	0.501	−0.008	NR
Diastolic blood pressure	0.405	0.047	−0.130	NR
Forced expiratory volume	−0.626	−0.535	NR	NR
White blood cell count	−0.115	−0.020	0.021	NR
Red blood cell count	−0.367	−0.096	NR	NR
Hemoglobin	−0.299	0.261	−0.246	NR
Hematocrit	−0.435	−0.036	NR	NR
C-reactive protein (log)	NR	0.122	NR	0.0954
Albumin	−0.310	−0.220	−0.236	−0.0336
Lymphocyte (%)	NR	−0.033	NR	0.0120
Alkaline phosphatase	−0.333	0.218	0.081	0.00188
Creatinine	0.181	0.148	0.142	0.0095
Blood glucose	0.129	NR	0.036	0.0195

NR was given for biomarkers that were “not reported” in the primary literature.

## DISCUSSION

### Phenotypic age, health-status, and mortality

Biological aging measurements using clinically observable data (i.e., phenotypes) have produced robust estimations and predictions of aging-related outcomes and mortality [41]. Much of recent biological clock research has focused on methylation data, but phenotypic features also offer powerful mortality and aging predictive power [35]. Phenotypic variables offer benefits at multiple levels in that they provide crucial insights into the physiological state of the subject in addition to providing an aggregate measure, albeit indirect, of the changes in various hallmarks of aging. Perhaps most importantly, changes in these phenotypic biomarkers are mechanistically linked to organ and cellular functions and, by extension, health outcomes and health span. Most of these phenotypic biomarkers are also highly actionable with lifestyle and dietary changes within a reasonably short period of time. Finally, they are much easier to collect than molecular measures due to lower cost and technology barriers. This suggests that phenotypic clocks could be easier to scale than epigenetic clocks since these measures are routinely collected in clinical settings at relatively affordable cost and the health benefits of tracking one’s phenotypic age are easily understood both by the individual and the healthcare system.

### Epigenetic clock associations with health and mortality

Epigenetic clocks have been shown to be significantly associated with various deleterious phenotypes. Multiple epigenetic clocks have found that body mass index (BMI) is correlated with increased epigenetic age, but further research is needed to better understand this relationship [51, 52]. To our knowledge, only one longitudinal study has found obesity to be the cause, rather than a consequence, of increased epigenetic age [53]. Multiple clocks found that high levels of alcohol intake were associated with increased epigenetic age. However, moderate levels of alcohol intake were not associated with increased epigenetic age, suggesting a non-linear relationship. Other disease states associated with elevated biological age include HIV [54–57], chronic obstructive pulmonary disease (COPD) [41, 58, 59], schizophrenia [60], post-traumatic stress disorder (PTSD) [61], smoking [62–67], particulate matter [68–70], diabetes [71–75], frailty [76] and socioeconomic status [77]. Recently, Noroozi et al. (2023) [96] identified several lifestyle and socio-economic variables impacting epigenetic aging rates, including sleep quality, education level, yoga practice, and more.

### Epigenetic clocks *in vitro* vs. *in vivo*

A key advantage of epigenetic clocks is their ability to provide robust aging estimations across tissues, physiological systems, and life stages. Unlike phenotypic clocks, epigenetic clocks are able to measure cellular changes both *in vitro* and *in vivo*. Additionally, they are able to generalize across tissues [20], making them particularly useful in both clinical and research settings. Furthermore, epigenetic clocks have been shown to provide insights into systemic physiological changes using only blood samples [97]. A recent clock, SystemsAge [97], proved that single blood DNA methylation tests have the ability to capture heterogeneous aging patterns across physiological systems. Their findings showed that providing scores for each physiological system can more accurately capture disease risk, better facilitating personalized care plans compared to a single global aging metric.

### Dimensionality reduction

Many biological clocks have utilized dimensionality reduction for a variety of reasons. First, methylation data is highly dimensional, with the common 450k arrays producing over 450,000 features. Additionally, high levels of entropy can often be present in methylation data due to various causes, including sample preparation, beads per CpG, batch effects, and probe chemistry and hybridization issues [80–84]. Dimensionality reduction can reduce noise caused by such deviations because entropy will likely not covary across features. Lastly, dimensionality reduction can provide significant improvements in computational tractability. The most common dimensionality reduction method used in extant biological clock literature is Principal Component Analysis (PCA) [85, 86, 49]. PCA is a computationally tractable linear dimensionality reduction approach and has proven to increase the accuracy of predictions in a variety of cohorts. In recent years, various non-linear dimensionality reduction methods have been proposed, including Isometric Mapping (Isomap), t-Distributed Stochastic Neighbor Embedding (t-SNE), and Unified Manifold Approximation and Projection (UMAP) [87]. To date, only one biological clock (DeepMAge) has utilized these more complex, non-linear approaches [21]. The authors attained state-of-the-art performance, but additional research is needed to address whether predictive improvements are significant enough to warrant the increased computational expense of non-linear dimensionality reduction techniques.

### Limitations

The main limitation of this review is the inability to objectively measure performance across heterogeneous cohorts and environments. Recent research has provided such objective comparisons [88–91]. This review set out to instead provide a comprehensive layout of extant research into two disparate but related fields: epigenetic clocks and phenotypic clocks. Additionally, this review is limited by its exclusion criteria, namely mitotic clocks and clocks that were built using non-human subjects. Consequently, this review did not seek to provide objective statistics to measure the performance of various methods. Lastly, this review was limited by its exclusion of non-English publications.

## CONCLUSIONS

Despite a recent surge in biological clock research, best practices are still empirical. Epigenetic clocks have illustrated superior chronological age estimation capabilities, but their ability to provide insights into mortality and disease has been shown to be moderate. In recent years, epigenetic clocks built using neural networks have attained state-of-the-art performance but must be paired with interpretability approaches such as SHAP [92] to understand the “black box” nature of the models. Phenotypic clocks have shown to be better predictors of mortality than chronological age and do so using easily measurable clinical variables. Since methylation array technology is still relatively cost-prohibitive in clinical or hospital settings, phenotypic clocks may provide the most utility in the short term.
